# Characterization of a novel polyvinyl alcohol/chitosan porous hydrogel combined with bone marrow mesenchymal stem cells and its application in articular cartilage repair

**DOI:** 10.1186/s12891-019-2644-7

**Published:** 2019-05-29

**Authors:** Liangquan Peng, Yong Zhou, Wei Lu, Weimin Zhu, Yusheng Li, Kang Chen, Greg Zhang, Jian Xu, Zhenhan Deng, Daping Wang

**Affiliations:** 1 Department of Sports Medicine, the First Affiliated Hospital of Shenzhen University, Shenzhen Second People’s Hospital, Shenzhen, 518035 Guangdong China; 2 School of Medicine, Shenzhen University, Shenzhen, 518060 Guangdong China; 3 Clinical College of Anhui Medical University Affiliated Shenzhen Second Hospital, Shenzhen, 518035 Guangdong China; 4 Key Laboratory of Tissue Engineering of Shenzhen, Shenzhen Second People’s Hospital, the First Affiliated Hospital of Shenzhen University, Shenzhen, 518035 Guangdong China; 5 Guangzhou Medical University, Guangzhou, 510182 Guangdong China; 6 Department of Orthopaedics, Xiangya Hospital, Central South University, Changsha, 410008 Hunan China; 7 McGovern Medical School, University of Texas Health Science Center at Houston, Houston, TX 77054 USA

**Keywords:** Hydrogel, Polyvinyl alcohol, Chitosan, Bone mesenchymal stem cells, Cartilage repair, Tissue engineering

## Abstract

**Background:**

Different substances are combined to compensate for each other’s drawbacks and create an appropriate biomaterial. A novel Polyvinyl alcohol (PVA)/chitosan (CS) porous hydrogel was designed and applied to the treatment of osteochondral defects.

**Methods:**

Hydrogels of various PVA/CS ratios were tested for physiochemical and mechanical properties in addition to cytotoxicity and biocompatibility. The hydrogels with the best PVA/CS ratio were used in the animal study. Osteochondral defects were created at the articular cartilage of 18 rabbits. They were assigned to different groups randomly (*n* = 6 per group): the osteochondral defect only group (control group), the osteochondral defect treated with hydrogel group (HG group), and the osteochondral defect treated with hydrogel loaded with bone marrow mesenchymal stem cells (BMSCs) group (HG-BMSCs group). The cartilage was collected for macro-observation and histological evaluation at 12 weeks after surgery.

**Results:**

The Hydrogel with PVA/CS ratio of 6:4 exhibited the best mechanical properties; it also showed stable physical and chemical properties with porosity and over 90% water content. Furthermore, it demonstrated no cytotoxicity and was able to promote cell proliferation. The HG-BMSCs group achieved the best cartilage healing.

**Conclusions:**

The novel PVA/CS porous composite hydrogel could be a good candidate for a tissue engineering material in cartilage repair.

**Electronic supplementary material:**

The online version of this article (10.1186/s12891-019-2644-7) contains supplementary material, which is available to authorized users.

## Background

Articular cartilage (AC) is a tissue that is notorious for its low regenerative capacity. Traumatic AC lesions often heal poorly and typically lead to cartilage degeneration and osteoarthritis (OA) [1, 2]. In fact, the AC rarely heals when the diameter of chondral defect exceeds 3 mm [3]. Among all the existing therapeutic options for the chondral defect, conventional management techniques, such as microfracture and mosaicplasty, usually end up with unsatisfactory outcomes [4]. Recently, many new treatment methods have been created, such as graft, periosteum, perichondrium, allographic chondrocyte transplantation, and so on [5].

Tissue engineering, by using a combination of cell seeding, scaffold, and cytokines, represents the most promising way of curing cartilage injury [6]. Extensive efforts have been devoted to searching for appropriate biomaterials and polymer-based scaffolds in tissue engineering strategies for cartilage repair. Hydrogels have been used as a promising biomaterial in many biomedical applications such as drug delivery and tissue engineering in recent decades [7]. Polyvinyl alcohol (PVA) is a synthetic, biocompatible polymer that has been well studied for use in bioengineered tissue scaffolds due to its relatively high strength, creep resistance, water retention, and porous structure [8]. Chitosan (CS) is a linear, semi-crystalline and biocompatible polysaccharide. One of CS’s most promising features is its excellent ability to be processed into porous structures in cell transplantation and tissue regeneration [9]. A scaffold that’s made of a single polymer does not have all the properties that are desirable in biomaterials. Therefore, a combined material may solve this problem. Moreover, concerns about cell division, proliferation, and migration, as well as biodegradability continue to remain unsolved [10–12].

Polyvinyl alcohol/chitosan (PVA/CS) based hydrogels blends have a high blood compatibility [13] and are good candidates for use as matrices for the controlled delivery of drugs [14]. Koyano et al. reported that PVA/CS hydrogels blended in an autoclave without the use of crosslinkers such as glutaraldehyde or paraformaldehyde exhibited high attachment to and promoted growth of cultured fibroblast cells depending on the CS content [15]. Yang et al. used a combinational method to crosslink the PVA/CS hydrogel membrane, which significantly improved its swelling, thermomechanical stability, anti-bacterial properties, and decreased water evaporation [16]. Though the PVA/CS hydrogel has achieved good results when applied to the treatment of AC defects, the mechanical properties and biocompatibility were not well studied, and the best ratio of PVA/CS hydrogel to be used as the scaffold during AC repair remained unclear [17, 18].

In this study, the natural materials PVA and CS were mixed in different proportions; this can adjust the mechanical properties and biocompatibility of the hydrogel in addition to overcoming the shortcomings of the single material hydrogel. This novel PVA/CS porous hydrogel was tested for its physical-chemical properties, mechanical performance, and in vitro culturing with rabbit bone marrow mesenchymal stem cells (BMSCs). The hydrogel alone and combining it with BMSCs were both applied in the treatment of cartilage repair and regeneration in a rabbit osteochondral defect model in order to validate the effects of hydrogel on the in vivo model.

## Methods

### Isolating and culturing of rabbit BMSCs

The animal experiment was carried out in accordance with relevant guidelines and regulations, and was approved by the Medical Ethics Committee of the First Affiliated Hospital of Shenzhen University (Grant number: LL2015028). The entirety of the procedure was conducted in sterile conditions. The bilateral femurs with all the soft tissue removed were collected from mature male New Zealand White Rabbit (2.5 ± 0.3 kg, 6 months old, provided by Guangdong Medical Laboratory Animal Center), and immersed in sterile phosphate buffer saline (PBS) solution. The femoral cavity was flushed with Dulbecco’s modification of Eagle’s medium (DMEM) culture solution repeatedly. Then, the flushing solution was collected and centrifuged at 1800 rpm at room temperature (RT) for 7 min. After aspiration of the supernatant, the cell sediment was resuspended, and the supernatant was aspirated again using the same procedure. Then cells were resuspended and dispersed by gentle pipetting in culture medium, composed of 80% mesengro (StemRD Ltd., USA) + 10% medium supplement (StemRD Ltd., USA) + 10% fetal bovine serum (FBS, Thermo Fisher Scientific Ltd., USA) + 0.1% basic fibroblast growth factor (FGF, Thermo Fisher Scientific Ltd., USA) + 1% Penicillin-Streptomycin (P/S, Hyclone Ltd.,USA) + 0.1% amikacin (Thermo Fisher Scientific Ltd., USA). The cells were inoculated on a 10cm^2^ culture dish in the controlled conditions of 37 °C and 5% CO_2_. The medium was changed every 3 to 4 days. Once 70 to 80% confluence was reached, the cells were detached with 0.25% trypsin-EDTA (Life Technologies Ltd., USA) and passaged. The second passage cells were used in the experiment, while the fourth passage cells were maintained as a backup.

### Composite hydrogel preparation

**Fig. 1:**
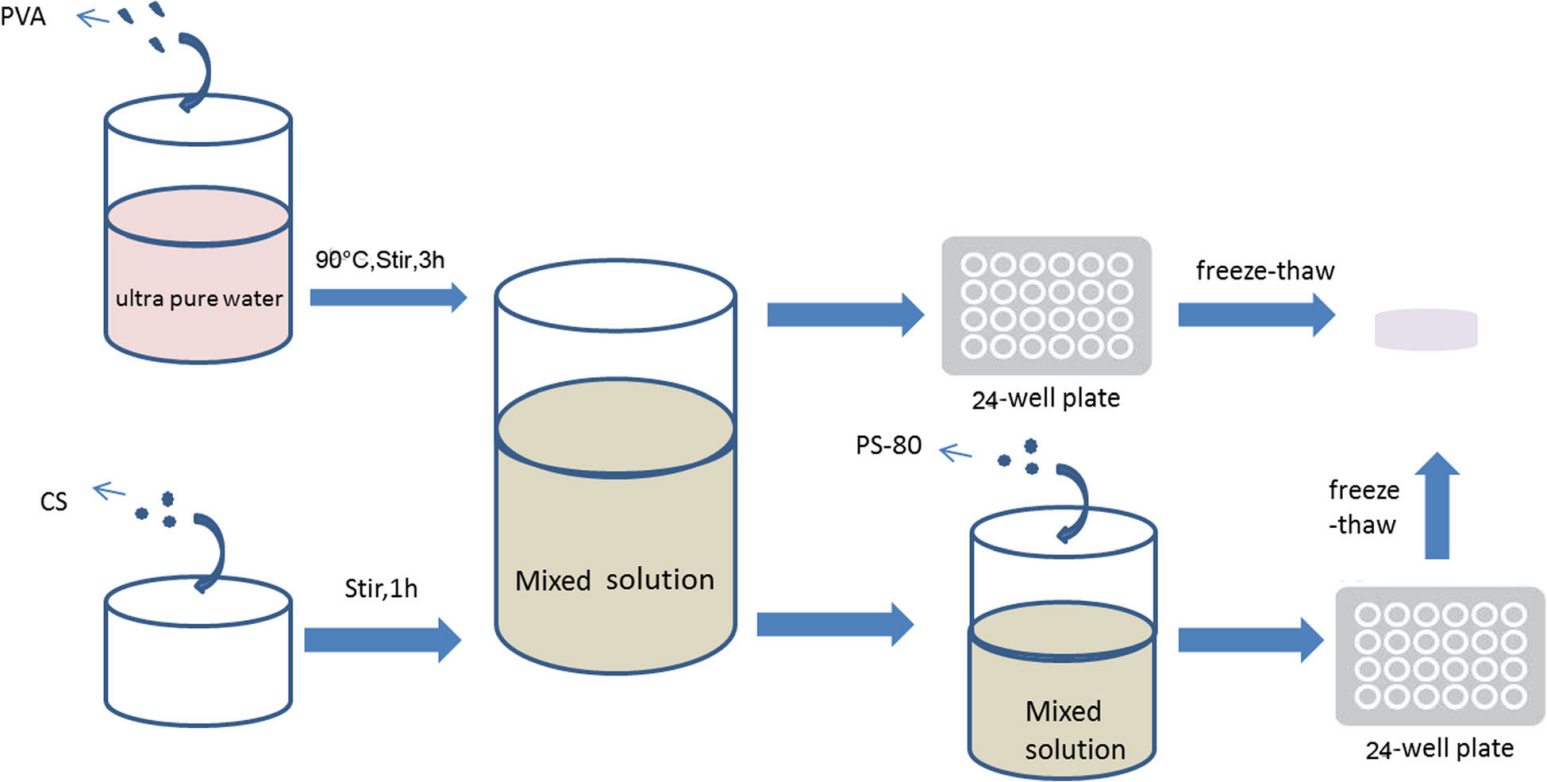
Diagram of PVA/CS composite hydrogel preparation

The hydrogel used in this study was prepared using a method of freeze-thaw cycles (Fig. 1) [19]. PVA, CS, polysorbate (PS)-80, DMEM cell culture medium, fetal bovine serum, and lyophilizer (solution) were purchased from Sigma Aldrich Ltd., Shanghai, China. 5 g PVA was discharged into ultra-pure water and swelled after heating in 60 °C for 20 min. The system was stirred in the magnetic blender for 3 h in 90 °C to ensure that the PVA was completely dissolved. The PVA solution was prepared with 10% weight ratio concentration. CS powder and 1% acetic acid (1 g: 50 ml) were poured into the beaker. The system was covered, sealed, and then blended on a magnetic blender for 1 h to achieve complete dissolution. The mixture of these two solutions at various proportions was left in the vacuum drier for 1 h and degassed. The mixture solution was added into a sterile 24-well plate, with each well containing less than 2 mL of solution. The plate was sealed and kept in − 20 °C for 20 h and then thawed for 8 h in RT. The freeze-thaw cycle was repeated seven times total. Afterwards, 1% sodium hydroxide (NaOH) solution was added into the 24-well plate to adjust pH to neutral. The mixture was then kept in ultra-pure water.

### Composite porous hydrogel preparation in the experimental group

PS-80, a surfactant, was added to the mixture of PVA and CS solution of various ratios mentioned in Table 1 at a weight ratio of 15:1 in the experimental groups, while the control groups with various PVA/CS ratio were left intact. After 30 min stirring, the solutions in the experimental groups underwent the freeze-thaw and neutralizing process as described above. The residual PS-80 on the surface of the hydrogel was cleared with ultra-pure water.

**Table 1:** Code of hydrogel of varying PVA/CS ratio

Control group (without PS-80)	Experimental group (with PS-80)	PVA/CS(W:W)
a	A	5:5
b	B	6:4
c	C	7:3
d	D	8:2
e	E	9:1

### BMSCs seeding on hydrogel

The hydrogel was sterilized under UV radiation and immersed in normal saline (NS) which was renewed every three hours for three times total. One day before the seeding, the hydrogel was immersed in complete medium and cultured in an incubator for 12 h. The second passage of BMSCs suspension was pipetted into a 24 wells plate with approximately 50,000 cells per well and cultured in incubator with the medium changed every 3 days.

### Chondrogenesis differentiation assay

The BMSCs were maintained under the chondrogenic induction medium (DMEM, 0.2 mM ascorbic 2-phosphate, 20% FBS, and 10 mM glycerol 2-phosphate) supplemented with 10 ng/ml transforming growth factor β3 (TGFβ3; R&D Systems, Minneapolis, MN). Chondrogenesis was evaluated using Alcian blue (Millipore, Billerica, MA, USA) staining [20, 21].

### Water content (WC) of hydrogel

Hydrogel pieces of equal size and weight were prepared, cleared of residue and weighed (W1); they were then desiccated in a vacuum drier for 48 h and weighed again (W2). WC was calculated as WC = (W1-W2)/W1 × 100%. This procedure was repeated three times.

### Swelling rate (SR) of hydrogel

Desiccated hydrogel weighing W2 was immersed in ultra-pure water for various durations: 1 h, 3 h, 7 h, 1d, 2d, 3d, 4d, 5d, 6d, and 7d. Afterwards, the weight of each hydrogel was recorded (Wt). SR was calculated as SR = Wt/W2. A curve was plotted to reflect SR changes over time.

### Differential scanning calorimetry (DSC)

The performance of PVA in the desiccated hydrogel was analyzed with differential scanning calorimetry. 5 mg hydrogel was put in the holder in nitrogen where the temperature is increased/decreased at a rate of 10 °C/min. The temperature range was set as 25–250 °C.

### Characterization of hydrogel

Infrared (IR) spectroscopy analysis of the vacuum-desiccated hydrogel was performed with a Nicolet 740 spectrometer. The morphology of the porous structure of vacuum-desiccated hydrogel was observed with a JEM-6360 scanning electron microscope (SEM). The average sizes and size distributions of the hydrogel were determined based on imaging of the particles using Nano Measurer 1.2 software.

### Mechanical property test

The mechanical performance of the hydrogel in all the groups was analyzed with an electro-mechanical universal tester. A hydrogel cylinder of 12 mm diameter and 8 mm height was placed between the grips, and compression was applied at the rate of 2 mm/min. The compressive strength and tensile strength were measured. 5 samples were tested for each group.

### Live/dead cell viability assay

The hydrogel loaded with BMSCs underwent live/dead cell staining (Weikai Biological Ltd., China) at 1, 3, and 7 days after inoculation for observation of cell numbers and viability via a fluorescence microscope. Live cells were stained green while dead cells were red. Three wells were assigned to each sample.

### Cell counting kit-8 (CCK-8) assay

The hydrogel loaded with BMSC underwent cell proliferation assay at 1, 3 and 7 days after inoculation. Solution A was prepared by mixing CCK-8 reagent (Biyuntian Ltd., China) and culture medium with a volume ratio of 1:10. 400 μl of solution A was then added to the plate after removal of the old medium while protected from light. The system was incubated for 4 h to obtain solution B. 100 μl of solution B was added into each well in an empty 96-well plate, and the absorbance was measured by a micro-plate reader at 450 nm. Three measurements were performed for each sample.

### Rabbit osteochondral model

The hydrogel with the best physicochemical, biological, and mechanical properties was used in the animal study. 18 male mature New Zealand White Rabbits (2.5 ± 0.5 kg, 6 months old) were randomly distributed into three groups (*N* = 6 per group): the osteochondral defect only group (control group), the osteochondral defect treated with hydrogel group (HG group), and the osteochondral defect treated with hydrogel loaded with BMSCs group (HG-BMSCs group). The osteochondral surgery was performed randomly at the patellar groove of one hind limb of each rabbit. The surgical procedures employed have been reported before [22]. Under general anesthesia, shaving, disinfection, and draping were briefly performed before incision. A longitudinal skin incision of 30 mm was made and a medial parapatellar approach was used to perform a medial knee capsulotomy. An osteochondral defect of 4.5 mm diameter was made down to the subchondral layer with a tailored-made 4.5 mm trephine at the patellar groove of the femur. For the HG-BMSCs and HG groups, patches of hydrogel with and without BMSCs respectively were trimmed to fit the defect area and planted to the defect site. A sterile bio-protein sealant was applied to firmly attach the patch to the cartilage surface. All procedures were carefully performed; the tools were cooled with water to protect the cartilage and bone from heat damage. All procedures were performed by a single surgeon (LQ.P). All rabbits were caged separately after surgery and allowed to walk without restrictions.

### Macroscopic appearance

All rabbits were euthanized by air embolism at 12 weeks after the surgical procedure. The macroscopic appearance was observed right after sacrifice and evaluated by a blinded investigator (Y.Z). The International Cartilage Repair Society (ICRS) macroscopic cartilage evaluation score and grading (grade I, “normal”: 12; grade II, “nearly normal”: 11 to 8; grade III, “abnormal”: 7 to 4; grade IV, “severely abnormal”: 3 to 0) was used to assess all cartilage repairs [23].

### Histology

The repair tissues from the three groups were fixed in 10% neutral buffered formalin (NBF) for 48 h. The samples were then decalcified and paraffin embedded. Sections were cut at 5 μm thickness using a microtome, deparaffinized through xylene, and hydrated via ethanol gradient and water. Hematoxylin and eosin (H&E) staining was performed to reveal the morphology. Deparaffinized slices were dyed with hematoxylin for 5 min, given a 1 min water soak, differentiated with 1% hydrochloric acid ethanol for 30s, given a 15 min water soak, then dyed with 0.5% eosin for 3 min, given a distilled water soak, and finally sealed for observation after dehydration.

Safranin-O/fast green staining (IHC World; http://www.ihcworld.com/_protocols/special_stains/safranin_o.htm) was modified by extending the Safranin O step to 30 min and was performed to detect the proteoglycan and glycosaminoglycan matrices. Deparaffinized slices were dyed with Weigert’s iron hematoxylin working solution for 10 min; washed for 10 min; stained with fast green solution for 5 min; rinsed quickly with 1% acetic acid solution and stained in 0.1% Safranin O solution for 30 min; dehydrated and cleared with 95% ethyl alcohol, absolute ethyl alcohol, and xylene; and finally, mounted using resinous medium. The AC damage shown in the Safranin O staining images was further evaluated using the Mankin scoring system [24]. A higher score indicates more severe cartilage degeneration.

Immunohistochemical analysis of collagen type II (Col2) was performed to detect chondrocyte-specific matrix Col2. Deparaffinized slices were treated with 2% hyaluronidase in PBS at RT for 30 min for antigen retrieval. The slices were then washed in PBS solution and blocked with 5% donkey serum. Primary antibody anti-Col2 antibody (1:400, ab34712, Abcam, USA) was then applied and incubated at 4 °C overnight. Endogenous peroxidase was blocked with 0.5% hydrogen peroxide (H_2_O_2_) in PBS before sections were incubated with the secondary antibody (goat-anti-rabbit, BA 1000, Vector Laboratories, Burlingame, CA, USA, 1:300 dilution) for 2 h at RT. Afterwards, each slide was rinsed with PBS and incubated in ABC reagents (PK 7200, Elite ABC kits, Vector Laboratories, USA) for 1 h at RT. Subsequently, the DAB color reaction (SK-4100, Vector Laboratories, USA) kit was used to reveal specific antigen-positive cells, and hematoxylin (H3404, Vector LaboratoriesVector Laboratories, USA) counterstaining was performed to stain nuclei. Finally, each slide was hydrated through an ethanol gradient, cleared with xylene, and mounted in xylene-based Cytoseal.

3–4 images were taken using 200× magnification, including the entire defect cartilage area in each section. The relative Col2-positive matrix percentage (brown color area) in the defect cartilage area was quantified using NIS software. The positive area of all images of each animal were summed up and normalized to a 200× field (200 × field area = 0.2831 mm2 for bright field images).

### Statistical analysis

Data were presented as mean ± SD. The software SPSS 16.0 (version 15.0 for Windows; SPSS Inc., Chicago, IL, USA) was applied for statistical analysis and management. The one-way analysis of variance (ANOVA), SNK-q, and Dunnett’s T3 were applied for comparisons of multi-sample means and heterogeneity of variance. Any difference with *p* < 0.05 was considered as statistically significant.

## Results

### Chondrogenesis differentiation assay

**Fig. 2:**
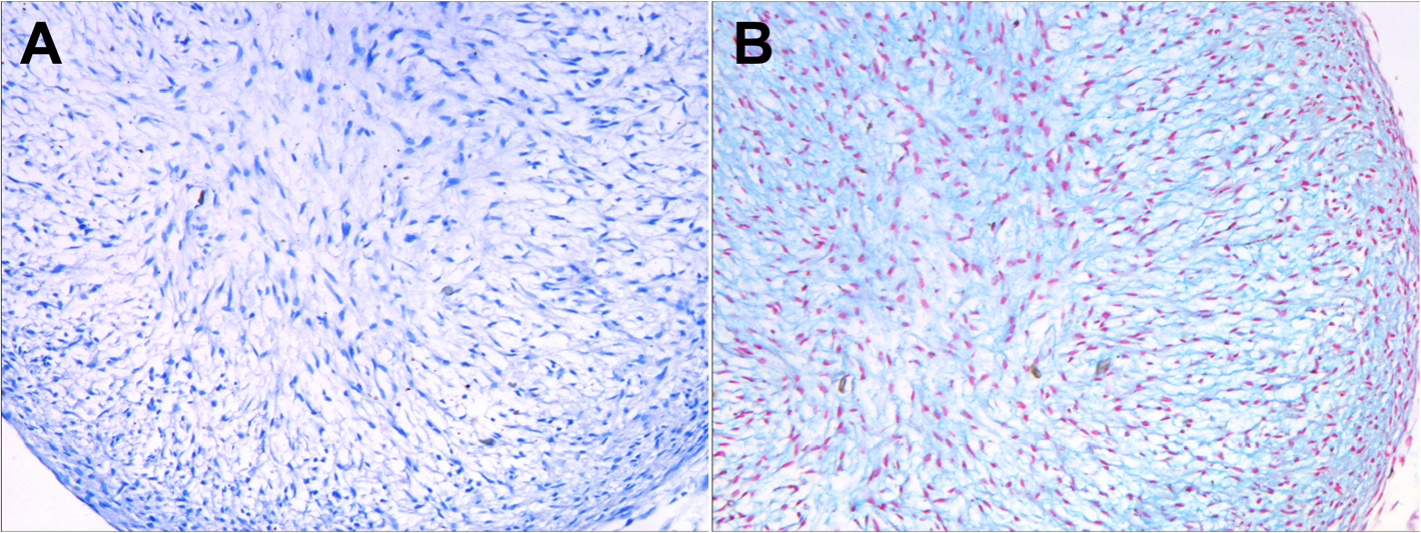
Chondrogenesis differentiation assay of the BMSCs after seeding on hydrogel (Alcian blue staining). **a** BMSCs seeded on hydrogel in normal cell culture medium. **b** BMSCs seeded on hydrogel in chondrogenic induction medium

Figure 2 showed the morphology of alcian blue staining of BMSCs seeded on hydrogel in normal cell culture medium and chondrogenic induction medium, respectively. A dramatic increase in chondrogenesis was detected in BMSCs seeded on hydrogel cultured with chondrogenic induction medium, indicating the potent capacity of BMSCs to differentiatd into chondrocytes when combined with the PVA/CS composite hydrogel (Fig. 2).

### Gross examination of PVA/CS composite hydrogel

**Fig. 3:**
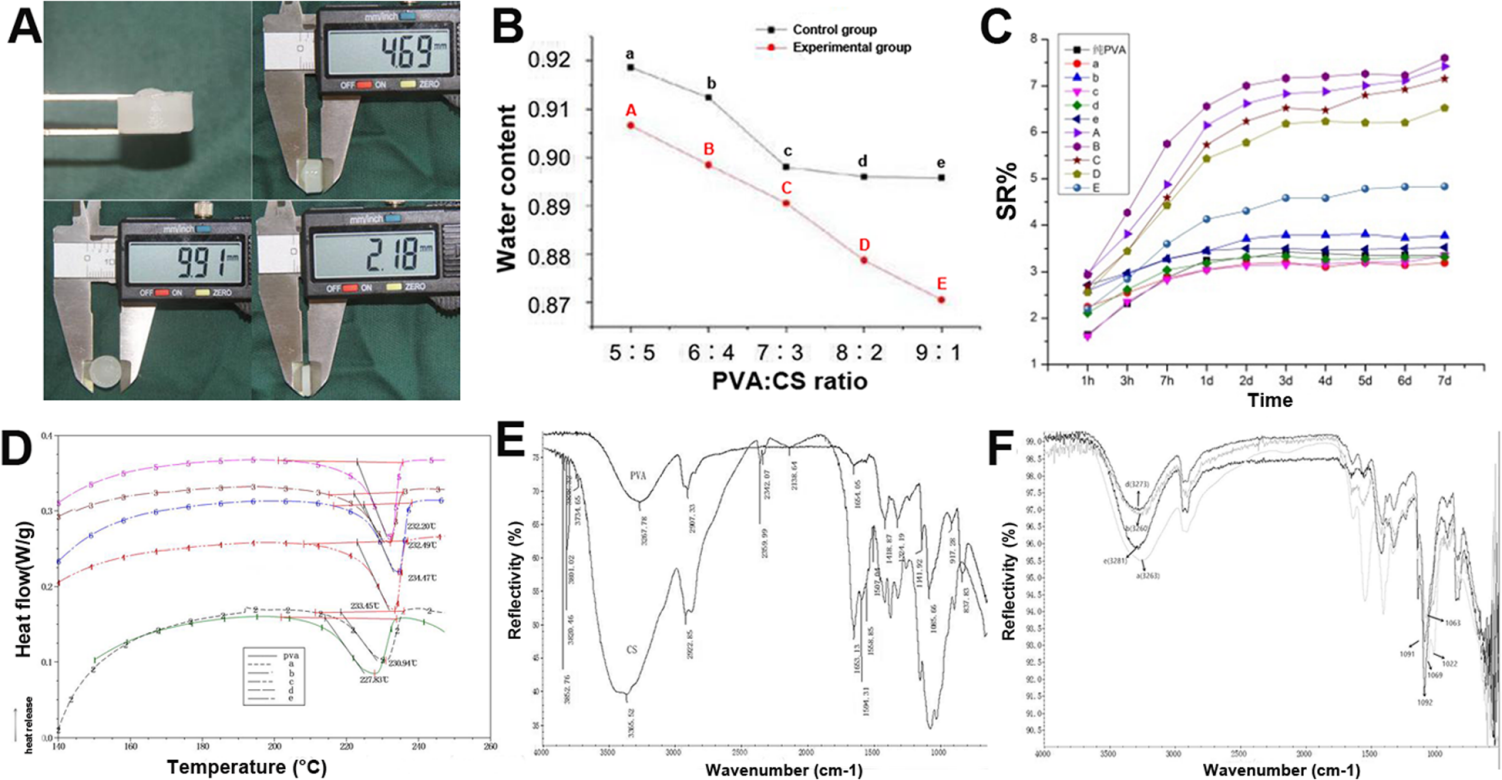
Evaluation of the physiochemical properties of the hydrogel of different PVA/CS ratios in the control and experimental groups. **a** Gross examination of PVA/CS composite hydrogel pieces of various thickness. **b** The WC of the hydrogel of varying PVA/CS ratios. **c** The SR of the hydrogel of varying PVA/CS ratios. **d** DSC curve of the hydrogel of varying PVA/CS ratios. **e** IR spectrum of pure PVA and CS. **f** IR spectrum of blends of PVA and CS of varying PVA/CS ratios

The hydrogel was the milky-white pieces with well-controlled thickness and diameter (Fig. 3a).

### Water content (WC)

According to the different codes of PVA/CS ratios (Table 1), three random samples similar in weight and size were taken from each mixture proportion to undergo WC analysis (Fig. 3b). The WC of all the samples studied was above 85%, and the WC decreased as the PVA proportion increased. The introduction of PS-80 decreased WC in all the samples regardless of their formula. The pair-wise comparison was only statistically significantly different between the groups E and e (*p* < 0.05). For intra-group comparisons, the difference between groups A and B (including group a and b) were not statistically significant (*p* > 0.05), while the differences among groups A, B, and C, D, and E (including group a, b, and c, d, e) were statistically significant (*p* < 0.05), indicating that the WC of the hydrogels in groups A and B (including group a and b) were the highest (Additional file 1: Table S1).

### Swelling rate (SR)

The SR of hydrogel holds the key to nutrient and waste exchange between the inside and outside of the human body [25]. The SR curve of various PAV/CS ratios was presented in Fig. 3c, indicating that the hydrogel swelled rapidly during the first 48 h regardless of the mix ratio, then slowed down gradually, and finally reached equilibrium on around day 5. We also found that the SR was positively correlated to PVA content, indicating that higher content of PVA was beneficial for retaining water. The experiment group had higher SR than the control group at each time point (*p* < 0.05) which may be attributed to emulsification, resulting in more pores formed within the hydrogel. Among the experimental groups, the SR in groups A and B were much higher than those in the other groups (*p* < 0.05), but there was no difference in SR when comparing group A with group B (*p* > 0.05). Therefore, the swelling performance of group A and group B is best in all the groups (Additional file 1: Tables S2, S3).

### Differential scanning calorimetry (DSC)

The performance of the final product of polymer blends depends on its miscibility, which is regarded as a key factor in developing new materials based on polymer blends [25, 26]. The glass transition temperature (Tg) of each sample is presented in Fig. 3d. The peak Tg of pure PVA was 227.83 °C; those in the control groups were 230.94 °C (group a), 232.49 °C (group b), 233.45 °C (group c), 232.20 °C (group d), and 234.47 °C (group e), indicating that PVA was miscible with CS and there was a slight increase in Tg as PVA content increased.

### Infrared (IR) spectroscopy

IR spectroscopy was performed on pure PVA and CS as well as the blends to examine whether any of them were lost to evaporation (Fig. 3e, f). The IR spectrum of pure CS features the iconic IR peaks of NHCOCH^3^ with amide I at 1653 cm^− 1^ and amide II at 1594 cm^− 1^. Meanwhile, the absorption peak at 3267 cm^− 1^ in the pure PVA waveform is attributed to the stretching vibration of -OH group. As PVA content increased, the iconic IR peaks of CS shifts towards the right. This could be explained by the hydrogen bonding between the OH or NH groups of PVA and the CS molecule. This result proved the presence of both materials and their successful blending.

### Scanning electron microscope (SEM)

**Fig. 4:**
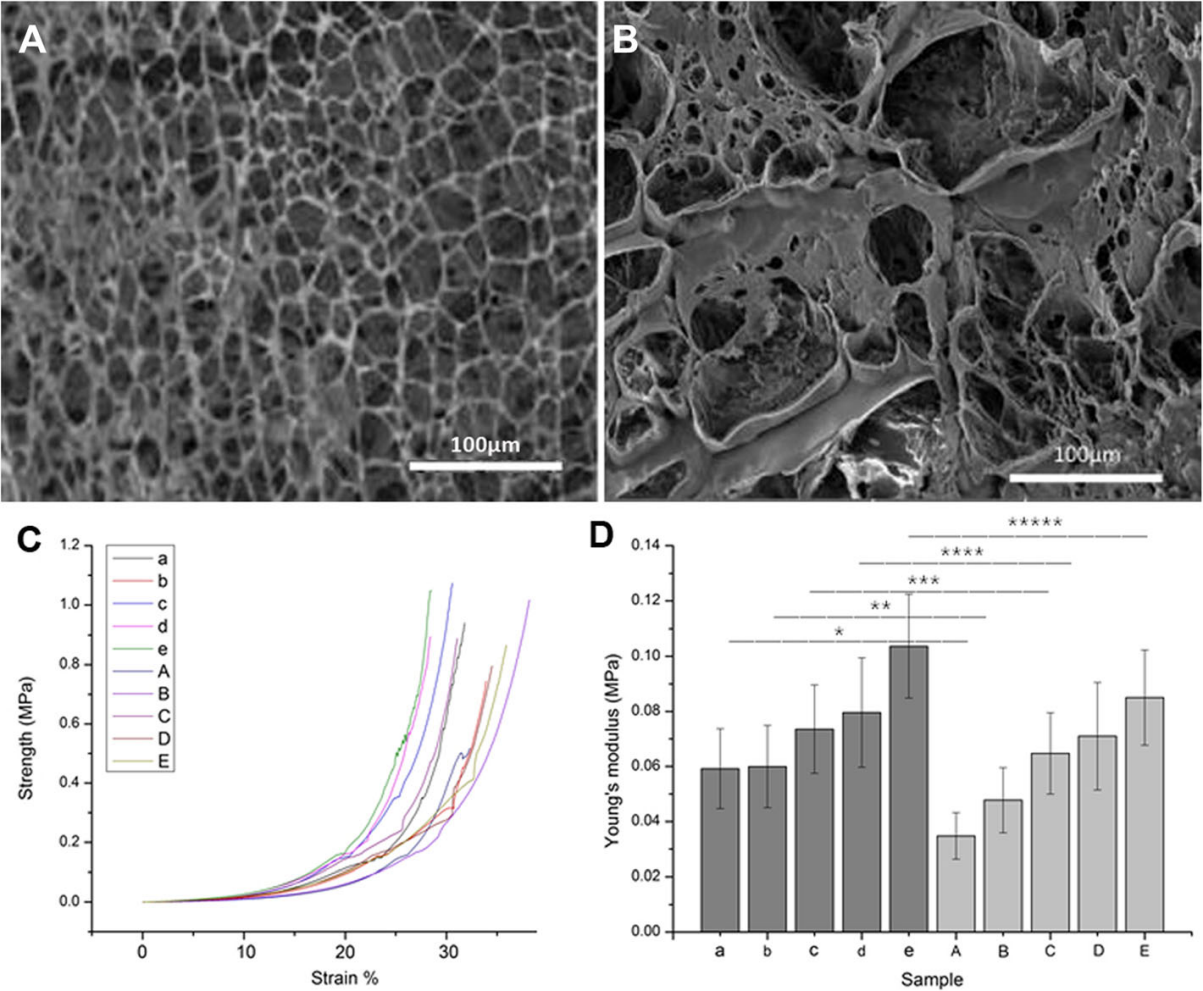
SEM images of the hydrogel micro-structure and Mechanical property test in the control and experimental groups. **a** SEM images of the hydrogel micro-structure in the control group. **b** SEM images of the hydrogel micro-structure in the experimental group. **c** Compressive-tensile strength curve. **d** Young’s modulus. a, b, c, d, e represent the stress-strain diagram and Young’s modulus of the control group a, b, c, d, e, and A,B,C,D,E represent the stress-strain diagram and Young’s modulus of the experimental groups. *,**,***,****: *p* < 0.05

Under the SEM, the structure of porous scaffold was formed in all the hydrogel; in addition, the pore size was consistent regardless of the different PVA/CS ratios in the control group. We could observe pores evenly distributed with size ranging from 20 to 100 μm in the control group (Fig. 4a). In contrast, the pore sizes (200 μm to 400 μm) were much larger and irregularly shaped in the experiment group (Fig. 4b). The space between large pores was filled with small pores and pores of all sizes were found on the surface and within the hydrogel structure. It has been reported that pore size around 300 μm is the most nourishing for cells [27]; in addition, pores varying in size are more conducive to cell adhesion and growth, as well as an exchange of nutrients and products from cells [28].

### Mechanical property

Figure 4c shows the compressive-tensile strength curve of the 10 composite hydrogel blends and Fig. 4d shows the Young’s modulus. The results showed that both Young’s modulus and compressive strength increased as the PVA content increased in both the experiment and control groups. For a particular PVA/CS ratio, mechanical performance was better in the control group than the experimental group (*p* < 0.05). The pair-wise comparison all showed significant statistical difference in the experimental groups (*p* < 0.05), and group E had the highest mechanical properties while group A had the lowest (Additional file 1: Table S4). However, if the mechanical properties of the hydrogel was too high, the shearing stresses created by the hydrogel after implantation would hamper the new tissue ingrowth aside the graft [29]. Therefore, choosing the best PVA/CS ratio should rely on taking other properties into consideration instead of being overly concerned with having the best mechanical properties.

### Live/dead cell staining

**Fig. 5:**
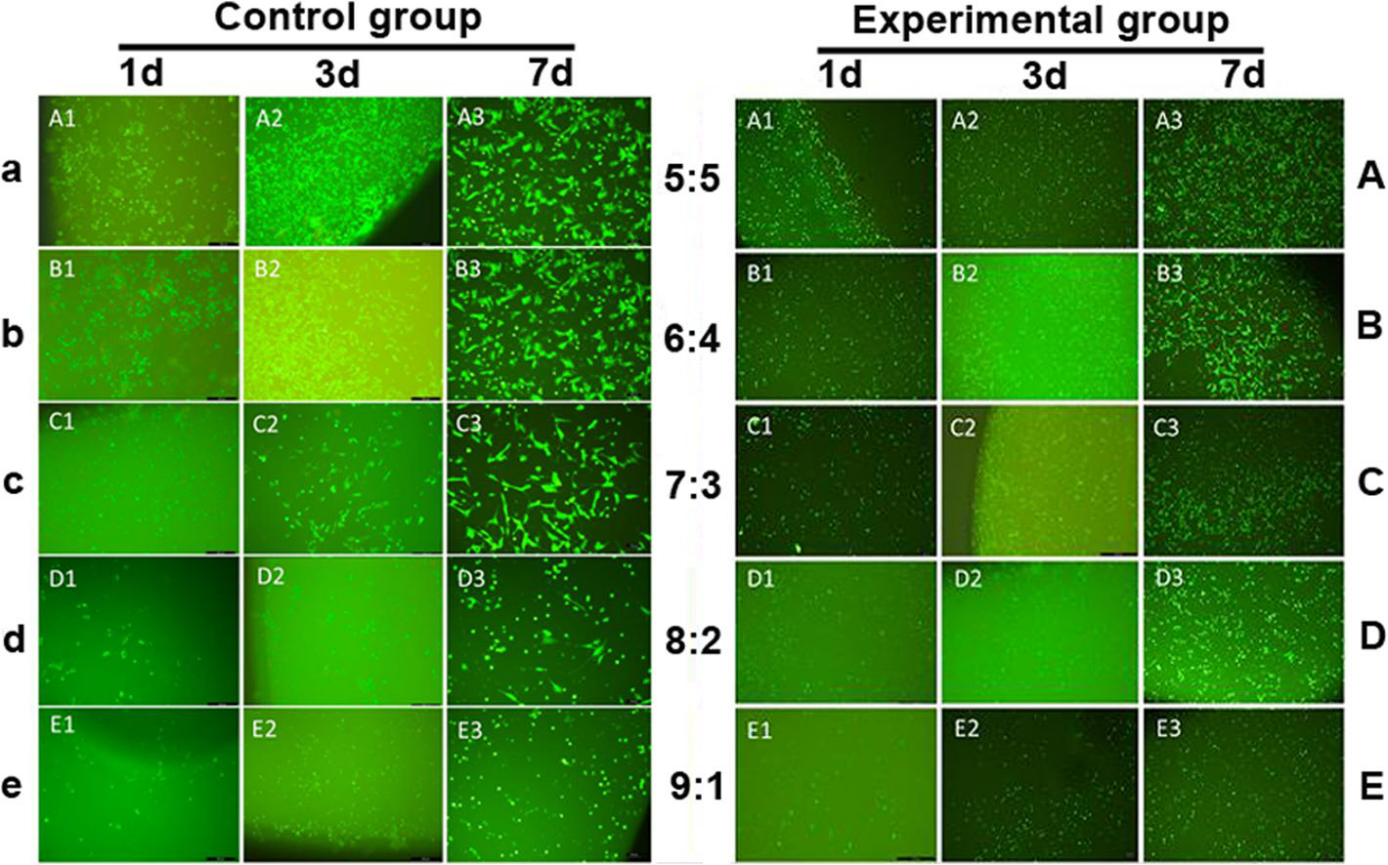
Images of live/dead cell staining at 1, 3 and 7 days after BMSCs loading in the control and experimental groups

On the first day after cell loading, the number of live cells decreased, and cells appeared spherical in all the groups. On day 3, cell numbers significantly increased in all groups. On day 7, the cells were in good shape, evenly distributed, and covered the entire surface of scaffolds in all groups. There were fewer cells, and cells transformed from polygonal to spherical as the PVA content increased in both experimental and control groups. More live cells that were evenly distributed were found in the experimental groups compared with the control groups (Fig. 5).

### CCK-8 cell proliferation assay

**Fig. 6:**
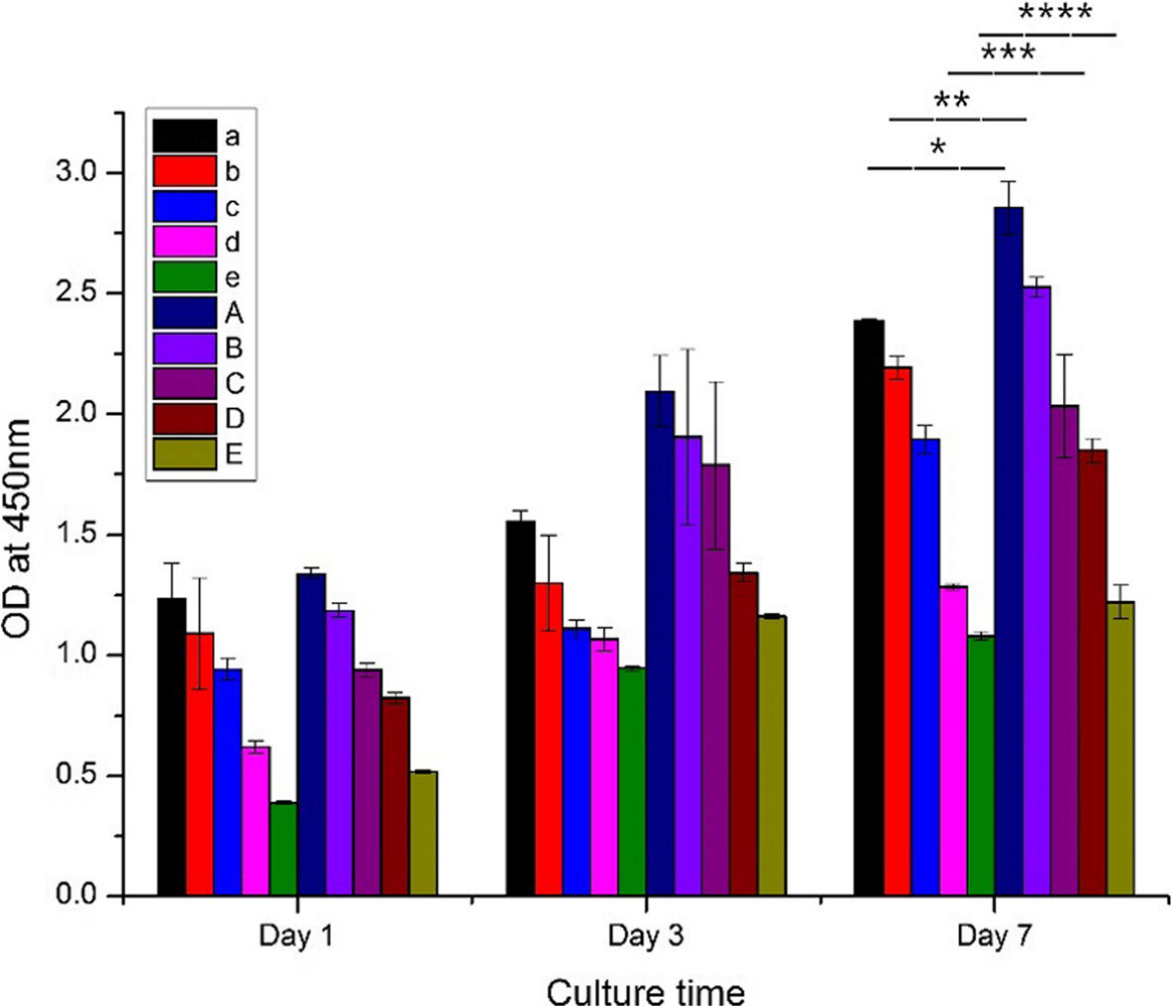
CCK-8 assay at 1, 3 and 7 days after BMSCs loading in the control and experimental group. *,**,***,****: *p* < 0.05

Cell growth and proliferation were evident in all the hydrogel blends (Fig. 6). The cells proliferated faster in the experiment groups than the control groups under the same conditions (*p* < 0.05), indicating that the hydrogel with higher porosity and larger pore diameter structure would accelerate cell proliferation. However, the cell proliferation rate was negatively correlated with the PVA content, reflecting the inferior biocompatibility of the synthetic materials compared with natural polymers (Additional file 1: Tables S5 and S6).

The hydrogel in group B with the PAV/CS ratio of 6:4 had better WC and SR than group C, D and E, and showed better mechanical properties than group A. It was considered to be the best graft as it showed good physicochemical, biological, and mechanical properties and was applied in the animal experiment.

### Macro evaluation of the osteochondral defect

**Fig. 7:**
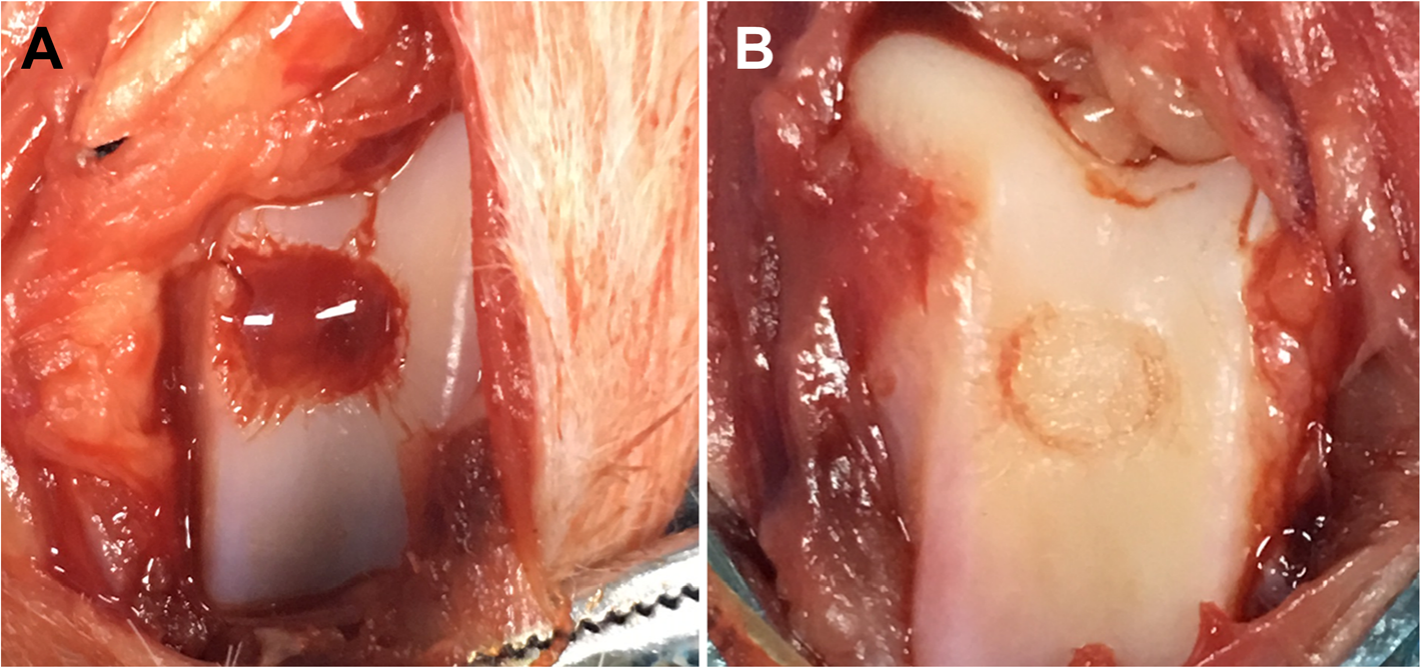
Macro observation of the osteochondral defect before (**a**) and (**b**) treatment in the HG-BMSCs group

The osteochondral defect before and after treatment in the HG-BMSCs group was shown in Fig. 7.

**Fig. 8:**
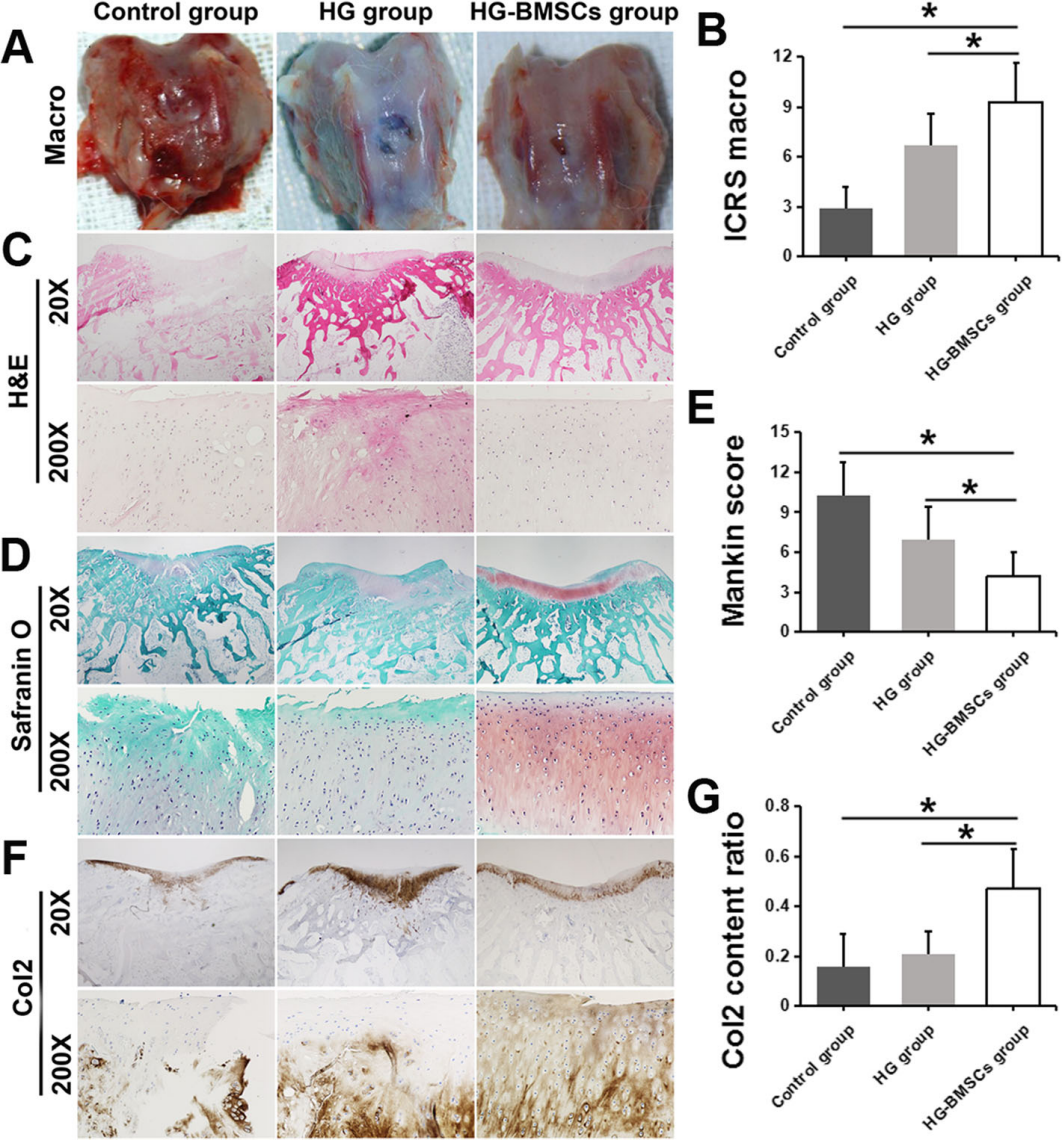
Macro and histological evaluation of the osteochondral defect healing in the control, HG and HG-BMSCs groups at 12 weeks after surgery. **a** The macro observation. **b** ICRS macroscopic assessment scoring. **c** H&E staining. **d** Safranin O staining, **e** Mankin Score. **f** Col2 staining. **g** Col2 content ratio quantification

At 3 weeks after surgery, we observed obvious osteochondral defect without any signs of healing in the control group. In the HG group, the defect area was characterized by a white, orderly, and even but incomplete repair surface. In the HG-BMSCs group, the defect area was repaired by the patch that attached to the surrounding normal cartilage seamlessly. The repaired AC features an orderly and even repair surface with homogeneous color and profile similar to a normal AC (Fig. 8a). In a subcategory of ICRS macroscopic assessment, integration to the border zone was significantly higher in HG-BMSC group than that in HG and control groups (9.3 ± 2.3 vs 6.7 ± 1.9 vs 2.9 ± 1.3, respectively; *p* < 0.05, Fig. 8b).

### Histological analysis

H&E and Saranin O staining also confirmed the superior morphology of AC repair in the HG-BMSCs group: hyaline-like cartilage healing with plenty of collagen matrix filling up the space beneath the even surface, leaving only a shallow unhealed gap. More Safranin O staining positive cartilage matrix containing proteoglycans and glycosaminoglycans could be observed at the injury site. In the control group, no signs of AC repair or matrix formation were observed except for a thin layer of fibers. The HG group had a mild AC repair effect; however, the repaired AC surface was incomplete and uneven (Fig. 8c, d). The Mankin score of AC cartilage in HG-BMSCs group (4.2 ± 1.8) was significantly lower than those in the control group (10.3 ± 2.4) and the HG group (6.9 ± 2.5), indicating better cartilage repair in the HG-BMSCs group (*p* < 0.05, Fig. 8e). The Col2 content ratio in the HG-BMSCs group (0.47 ± 0.16) was significantly higher than those in the control group (0.16 ± 0.13) and the HG group (0.21 ± 0.09), indicating that more Col2 regenerated in the HG-BMSCs group (*p* < 0.05, Fig. 8f, g).

## Discussion

The application of scaffolds in tissue engineering for treatment of cartilage injury could effectively accelerate cartilage healing and thereby avoiding complicated surgical procedures [30]. Although there is no ideal material, the scaffold used in in tissue engineering should be nonimmunogenic, nontoxic, biocompatible, biodegradable, cost-effective and easily tailored [31]. The cell-loaded hydrogel is favored as a synthetic material to be used in the chondral repair thanks to the advancement of cartilage tissue engineering [32]. The hydrogel is a promising way for repairing and regenerating damaged tissues by mimicking the structural and functional profile of the natural extracellular matrix (ECM). The hydrogel can promote the migration and differentiation of stem cells; moreover, it provides a free entrance for nutrients and oxygen as well as an avenue for cell excretion [33].

As an extensively utilized material in tissue engineering, CS is a positively charged and biodegradable glycosaminoglycan that shares a similar structure with natural mucopolysaccharide [34]. Its hydrophilic surface facilitates adhesion, proliferation, and differentiation of loading cells. It has become a major biomaterial by boasting low reaction to pathological inflammation, low risk of infection, low production of endotoxin, and good antibiosis [35]. Its mucopolysaccharide-like structure renders it to be universal in scaffold manufacturing [34, 35]. Autologous chondrocyte implantation (ACI) has long been used in the treatment of osteochondral defect and has achieved satisfied clinical outcomes; however, it was found that the implanted chondrocytes tend to differentiate into a fibroblast-like phenotype [36, 37]. Matrix-induced autologous chondrocyte implantation (MACI) techniques combined with scaffolds were designed to reduce and prevent the dedifferentiation of the chondrocytes during culture; they have attained good long-term outcomes to cure of chondral lesions in clinical trials [38, 39]. Most MACI scaffolds consist of type I collagen, type III collagen, or hyaluronic acid, which promote cartilage repair and regeneration of chondrocytes [40]. However, shortcomings of this procedure are also apparent. The procedure of MACI included several steps (chondrocyte harvest from the patient, expansion in vitro, and reimplantation), the need for reoperation of MACI is unavoidable. Other disadvantages include side effects to the donors and the poor regeneration ability of chondrocytes derived from the aged donors. Thus, hydrogels are designed to maintain the chondrogenic phenotype of chondrocytes. Buschmann et al. created an in-situ gelating CS based hydrogel that not only adhered to the defect area, but also retained chondrocytes’ phenotype and potential [41]. Accordingly, clinical trials have proved CS to be an effective cartilage repair treatment since 2015 [42].

PVA is a water-soluble crystal generated from the hydrolysis of polyvinylacetate. The use of PVA began in industry (food and drug especially) and commerce in the last century as an end product found in paint, resin, and food packaging. It is usually blended with other polymers, such as natural polymers and hydrophilic polymers [43]. As a type of hydrophilic polymer with good mechanical properties, fast degradation, and zero toxicity, PVA has been approved by FDA for use in the food and drug industry [43].

In our study, materials went through the freeze-thaw cycle and mixture with PS-80, becoming composite porous hydrogels of various PVA/CS mix ratios; these hydrogels were later analyzed for water content, swelling ratio, IR spectrum, DSC, SEM, mechanical properties, in vitro cytotoxicity and proliferation capacity. Through this series of steps, we screened the preferred type of hydrogel (hydrogel B) and loaded cells on the hydrogel, subsequently implanting it into the New Zealand White Rabbit. The cartilage damage healed with good results, validating the potential value of this new type of hydrogel. Asepsis was a key principle throughout the process of study, ensuring the study result was free from interference. Moreover, PS-80, used in this study, is favorable for cell proliferation as it increases porosity in the hydrogel. But since PS-80 itself is cytotoxic, we rinsed the hydrogel products several times to get rid of the residual PS-80. Under RT conditions, PVA is hardly soluble in water and tends to form a gel. Following the example of existing literature, we submerged PVA in warm water, allowing it to swell, and heating up the system to 90 °C while stirring the mixture. CS, on the other hand, is soluble in acid but insoluble in water. Therefore, it was dissolved in acid first. Once the gel was formed, the system was adjusted to neutral pH.

This study also presents some limitations. Firstly, to some extent, the emulsificante used in this study, PS-80, is cytotoxic and may interfere with the test results. Secondly, cartilage defects were not fully healed after three months, indicating this length of time may not be enough for chondral regeneration. Thirdly, we selected only several PVA/CS mix ratios. Building upon this study, we will explore more possibilities of mix ratios, take advantage of a less cytotoxic emulsificante, and extend the investigation into in vitro degradation and onto large animals of other species.

## Conclusions

The newly developed PVA/CS porous hydrogel shows high WC, good swelling capacity, stable physiochemical properties, well mechanical performance, low cytotoxicity, and excellently promotes cell adhesion and proliferation.

## Additional file

Additional file 1: **Table S1.** WC in the control group and experimental group with multiple comparisons. **Table S2.** SR of hydrogel in the experimental group. **Table S3.** SR of hydrogel in the experimental group with multiple comparisons. **Table S4.** Young’s modulus of mechanical properties evaluation in the experimental group with multiple comparisons. **Table S5.** CCK-8 cell proliferation in the experimental group. **Table S6.** CCK-8 cell proliferation in the experimental group Multiple Comparisons. (DOCX 27 kb)

## Data Availability

All data generated or analysed during this study are included in this published article.

## Abbreviations

AC: Articular cartilage
ANOVA: One-way analysis of variance
BMSCS: Bone marrow mesenchymal stem cells
CCK-8: Cell counting kit-8
Col2: Collagen type II
CS: Chitosan
DMEM: Dulbecco’s modification of Eagle’s medium
DSC: Differential scanning calorimetry
FBS: Fetal bovine serum
FGF: Fibroblast growth factor
H&E: Hematoxylin and eosin
ICRS: International Cartilage Repair Society
IR: Infrared
NaOH: Sodium hydroxide
NBF: Neutral buffered formalin
NS: Normal saline
OA: Osteoarthritis
P/S: Penicillin-Streptomycin
PBS: Phosphate buffer saline
PLGA: Poly lactic-co-glycolic acid
PS: Polysorbate
PVA: Polyvinyl alcohol
PVA/CS: Polyvinyl alcohol/chitosan
RT: Room temperature
SEM: Scanning electron microscope
SR: Swelling rate
Tg: Transition temperature
WC: Water content
