# Supplementary material for: Characterization of a novel polyvinyl alcohol/chitosan porous hydrogel combined with bone marrow mesenchymal stem cells and its application in articular cartilage repair
Source: BMC Musculoskelet Disord. 2019 May 29;20:257. doi: 10.1186/s12891-019-2644-7 (PMC6540438; doi:10.1186/s12891-019-2644-7)
Supplement: Supplementary file 1 — Table S1. WC in the control group and experimental group with multiple comparisons. Table S2. SR of hydrogel in the experimental group. Table S3. SR of hydrogel in the experimental group with multiple comparisons. Table S4. Young’s modulus of mechanical properties evaluation in the experimental group with multiple comparisons. Table S5. CCK-8 cell proliferation in the experimental group. Table S6. CCK-8 cell proliferation in the experimental group Multiple Comparisons. (DOCX 27 kb) [file 12891_2019_2644_MOESM1_ESM.docx]

**Table S1** WC in the control group and experimental group with multiple comparisons.

| Control group | | | | | Experimental group | | | | |
| --- | --- | --- | --- | --- | --- | --- | --- | --- | --- |
| (I)group | (J)group | Mean Difference (I-J) | Std.Error | Sig. | (I)group | (J)group | Mean Difference (I-J) | Std.Error | Sig. |
| a | b | .00614500 | .00366806 | .155 | A | B | .00807980 | .00401648 | .100 |
|  | c | .02056500* | .00366806 | .002 |  | C | .01603665* | .00401648 | .010 |
|  | d | .02254500* | .00366806 | .002 |  | D | .02776658* | .00401648 | .001 |
|  | e | .02277000* | .00366806 | .002 |  | E | .03603177* | .00401648 | .000 |
| b | a | -.00614500 | .00366806 | .155 | B | A | -.00807980 | .00401648 | .100 |
|  | c | .01442000* | .00366806 | .011 |  | C | .00795685 | .00401648 | .104 |
|  | d | .01640000* | .00366806 | .007 |  | D | .01968678* | .00401648 | .004 |
|  | e | .01662500* | .00366806 | .006 |  | E | .02795198* | .00401648 | .001 |
| c | a | -.02056500* | .00366806 | .002 | C | A | -.01603665* | .00401648 | .010 |
|  | b | -.01442000* | .00366806 | .011 |  | B | -.00795685 | .00401648 | .104 |
|  | d | .00198000 | .00366806 | .613 |  | D | .01172993* | .00401648 | .033 |
|  | e | .00220500 | .00366806 | .574 |  | E | .01999512* | .00401648 | .004 |
| d | a | -.02254500* | .00366806 | .002 | D | A | -.02776658* | .00401648 | .001 |
|  | b | -.01640000* | .00366806 | .007 |  | B | -.01968678* | .00401648 | .004 |
|  | c | -.00198000 | .00366806 | .613 |  | C | -.01172993* | .00401648 | .033 |
|  | e | .00022500 | .00366806 | .953 |  | E | .00826519 | .00401648 | .095 |
| e | a | -.02277000* | .00366806 | .002 | E | A | -.03603177* | .00401648 | .000 |
|  | b | -.01662500* | .00366806 | .006 |  | B | -.02795198* | .00401648 | .001 |
|  | c | -.00220500 | .00366806 | .574 |  | C | -.01999512* | .00401648 | .004 |
|  | d | -.00022500 | .00366806 | .953 |  | D | -.00826519 | .00401648 | .095 |

*p<0.05.

**Table S2** SR of hydrogel in the experimental group.

|  | Sum of Squares | df | Mean Square | F | Sig. |
| --- | --- | --- | --- | --- | --- |
| Between Groups | 27.598 | 4 | 6.899 | 88.967 | .000 |
| Within Groups | 1.939 | 25 | .078 |  |  |
| Total | 29.537 | 29 |  |  |  |

**Table S3** SR of hydrogel in the experimental group with multiple comparisons.

| (I)group | (J)group | Mean Difference (I-J) | Std.Error | Sig. |
| --- | --- | --- | --- | --- |
| A | B | -.25895472 | .16078078 | .120 |
|  | C | .29363387 | .16078078 | .080 |
|  | D | .88971775* | .16078078 | .000 |
|  | E | 2.43008066* | .16078078 | .000 |
| B | A | .25895472 | .16078078 | .120 |
|  | C | .55258859* | .16078078 | .002 |
|  | D | 1.14867246* | .16078078 | .000 |
|  | E | 2.68903538* | .16078078 | .000 |
| C | A | -.29363387 | .16078078 | .080 |
|  | B | -.55258859* | .16078078 | .002 |
|  | D | .59608387* | .16078078 | .001 |
|  | E | 2.13644679* | .16078078 | .000 |
| D | A | -.88971775* | .16078078 | .000 |
|  | B | -1.14867246* | .16078078 | .000 |
|  | C | -.59608387* | .16078078 | .001 |
|  | E | 1.54036292* | .16078078 | .000 |
| E | A | -2.43008066* | .16078078 | .000 |
|  | B | -2.68903538* | .16078078 | .000 |
|  | C | -2.13644679* | .16078078 | .000 |
|  | D | -1.54036292* | .16078078 | .000 |

*p<0.05.

**Table S4** Young’s modulus of mechanical properties evaluation in the experimental group with multiple comparisons.

| (I)group | (J)group | Mean Difference (I-J) | Std.Error | Sig. |
| --- | --- | --- | --- | --- |
| A | B | -.01411972* | .00166076 | .000 |
|  | C | -.02992289* | .00192820 | .000 |
|  | D | -.03618165* | .00164884 | .000 |
|  | E | -.05020801* | .00169492 | .000 |
| B | A | .01411972* | .00166076 | .000 |
|  | C | -.01580316* | .00191274 | .000 |
|  | D | -.02206193* | .00163072 | .000 |
|  | E | -.03608829* | .00167731 | .000 |
| C | A | .02992289* | .00192820 | .000 |
|  | B | .01580316* | .00191274 | .000 |
|  | D | -.00625877* | .00190240 | .001 |
|  | E | -.02028512* | .00194248 | .000 |
| D | A | .03618165* | .00164884 | .000 |
|  | B | .02206193* | .00163072 | .000 |
|  | C | .00625877* | .00190240 | .001 |
|  | E | -.01402635* | .00166551 | .000 |
| E | A | .05020801* | .00169492 | .000 |
|  | B | -2.68903538* | .16078078 | .000 |
|  | C | -2.13644679* | .16078078 | .000 |
|  | D | -1.54036292* | .16078078 | .000 |

*p<0.05.

**Table S5** CCK-8 cell proliferation in the experimental group.

|  | Sum of Squares | df | Mean Square | F | Sig. |
| --- | --- | --- | --- | --- | --- |
| Between Groups | 6.371 | 4 | 1.593 | 119.209 | .000 |
| Within Groups | .200 | 15 | .013 |  |  |
| Total | 6.571 | 9 |  |  |  |

**Table S6** CCK-8 cell proliferation in the experimental group Multiple Comparisons.

| (I)group | (J)group | Mean Difference (I-J) | Std.Error | Sig. |
| --- | --- | --- | --- | --- |
| A | B | .3288500* | .0817315 | .001 |
|  | C | .8214250* | .0817315 | .000 |
|  | D | 1.0051000* | .0817315 | .000 |
|  | E | 1.6348250* | .0817315 | .000 |
| B | A | -.3288500* | .0817315 | .001 |
|  | C | .4925750* | .0817315 | .000 |
|  | D | .6762500* | .0817315 | .000 |
|  | E | 1.3059750* | .0817315 | .000 |
| C | A | -.8214250* | .0817315 | .000 |
|  | B | -.4925750* | .0817315 | .000 |
|  | D | .1836750* | .0817315 | .040 |
|  | E | .8134000* | .0817315 | .000 |
| D | A | -1.0051000* | .0817315 | .000 |
|  | B | -.6762500* | .0817315 | .000 |
|  | C | -.1836750* | .0817315 | .040 |
|  | E | .6297250* | .0817315 | .000 |
| E | A | -1.6348250* | .0817315 | .000 |
|  | B | -1.3059750* | .0817315 | .000 |
|  | C | -.8134000* | .0817315 | .000 |
|  | D | -.6297250* | .0817315 | .000 |
